# Deeper Seated Than Skin Deep: Report of a Rare Case of Follicular Occlusion Tetrad and a Literature Review

**DOI:** 10.7759/cureus.39474

**Published:** 2023-05-25

**Authors:** Yilen K Ng-Wong, Blesset Alexander, Mery Bartl, Christine E Loftis, Dina Hammad, Emilia C Dulgheru, Mauricio De La Garza, Aidee C Nunez

**Affiliations:** 1 Internal Medicine, The University of Texas Rio Grande Valley, Edinburg, USA; 2 Rheumatology, University of Florida, Gainesville, USA; 3 Rheumatology, Doctors Hospital at Renaissance, Edinburg, USA; 4 Plastic and Reconstructive Surgery Institute, Doctors Hospital at Renaissance, Edinburg, USA; 5 Plastic Surgery, Doctors Hospital at Renaissance, Edinburg, USA

**Keywords:** pilosebaceous orifices, skin grafts, pilonidal cyst, dissecting cellulitis of scalp, acne conglobata, hidradenitis suppurativa, follicular occlusion

## Abstract

Follicular occlusion tetrad (FOT) is a clinical syndrome consisting of hidradenitis suppurativa (HS), acne conglobata (AC), dissecting cellulitis of the scalp (DCS), and pilonidal cyst (PC). These entities typically occur independently, but occasionally present simultaneously comprising FOT. The four components share similar pathophysiology affecting the apocrine glands, follicular hyperkeratinization being the hallmark of each entity. Understanding shared similarities of each disease is paramount for the treatment approach as the relapsing and chronic nature of this syndrome can be burdening to patients. We present the case of a 22-year-old obese Hispanic man with a history of tobacco use who presented with worsening skin lesions. The patient developed extensive facial cystic acne 5 years before presentation, followed by left axillary hidradenitis suppurativa lesions two years before the presentation and right axillary involvement one year after. Skin manifestations then expanded to include the lower back, gluteal and perineal areas. The patient was diagnosed with FOT and despite conservative medical management, his lesions failed to improve. He ultimately underwent multiple staged excisional debridement surgeries and skin grafts. Our case underlines the presence of a syndromic association of cutaneous lesions that share a common pathogenesis and emphasizes that this entity requires a multidisciplinary approach. New biologic therapies continue to emerge and may potentially prevent the need for surgical intervention and the burden associated with it.

## Introduction

Follicular Occlusion Tetrad (FOT) is a chronic inflammatory complex describing the simultaneous occurrence of hidradenitis suppurativa (HS), acne conglobata (AC), dissecting cellulitis of the scalp (DCS), and pilonidal cyst (PC). The four components share the same underlying pathophysiology affecting the apocrine glands.  As per Montes et al, all conditions are marked by follicular hyperkeratosis which leads to obstruction of the pilosebaceous orifices [1]. With continued obstruction, the sebum accumulates which leads to an increased risk of secondary infection. As this process evolves, communicating sinuses filled with purulent material are formed [2].   Smoking and obesity are modifiable risk factors in addition to genetic predisposition. FOT tends to have a recurrent and medically resistant course. Due to the paucity of cases reported, there are no treatment guidelines yet and management is often individualized requiring a multidisciplinary approach encompassing both medical and surgical specialties. As new biologic therapies emerge, the recognition of the syndrome becomes paramount. 

## Case presentation

**Figure 1:**
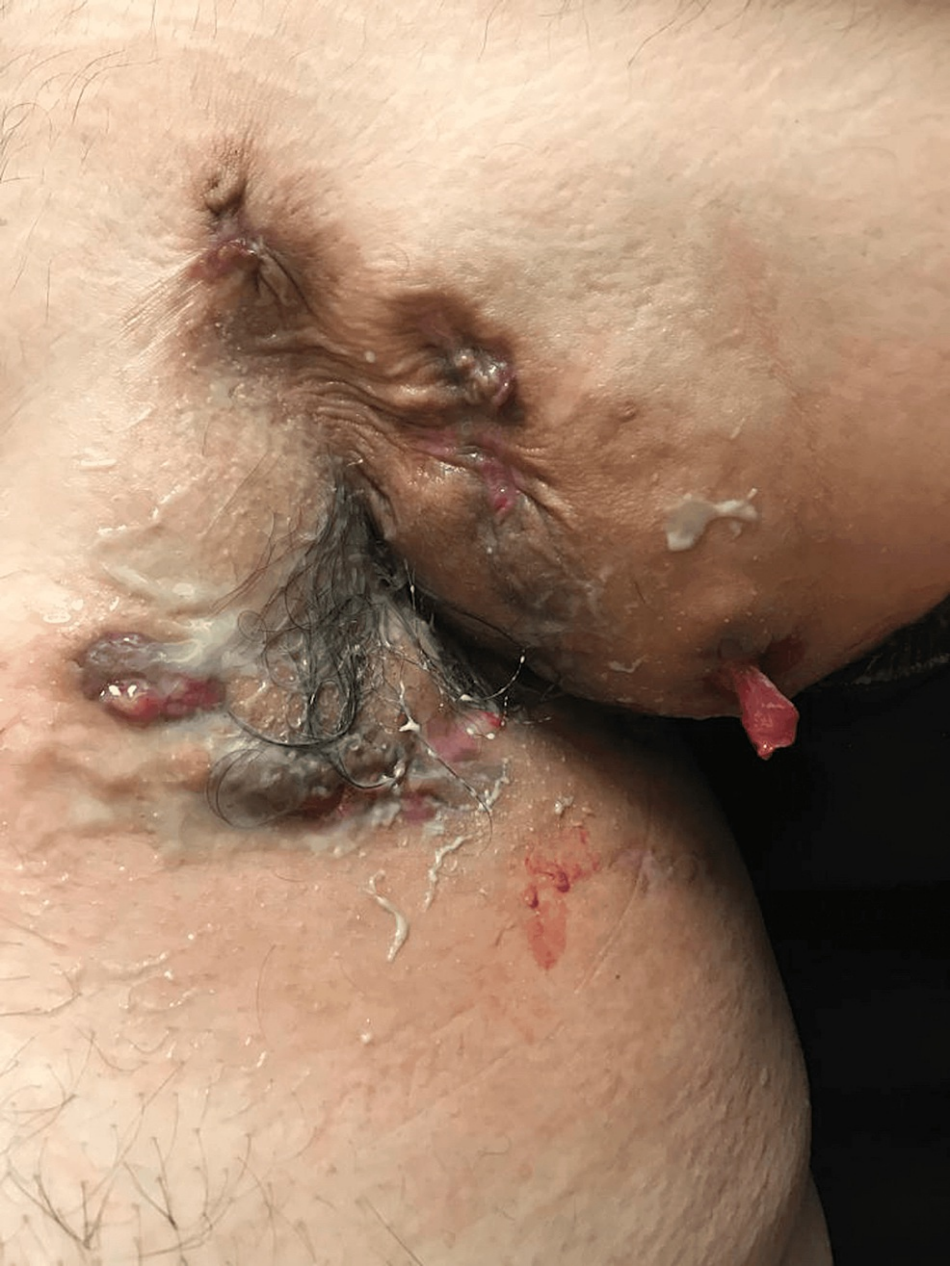
Hidradenitis suppurativa - purulent secretion and band-like scars in the left axillary region

**Figure 2:**
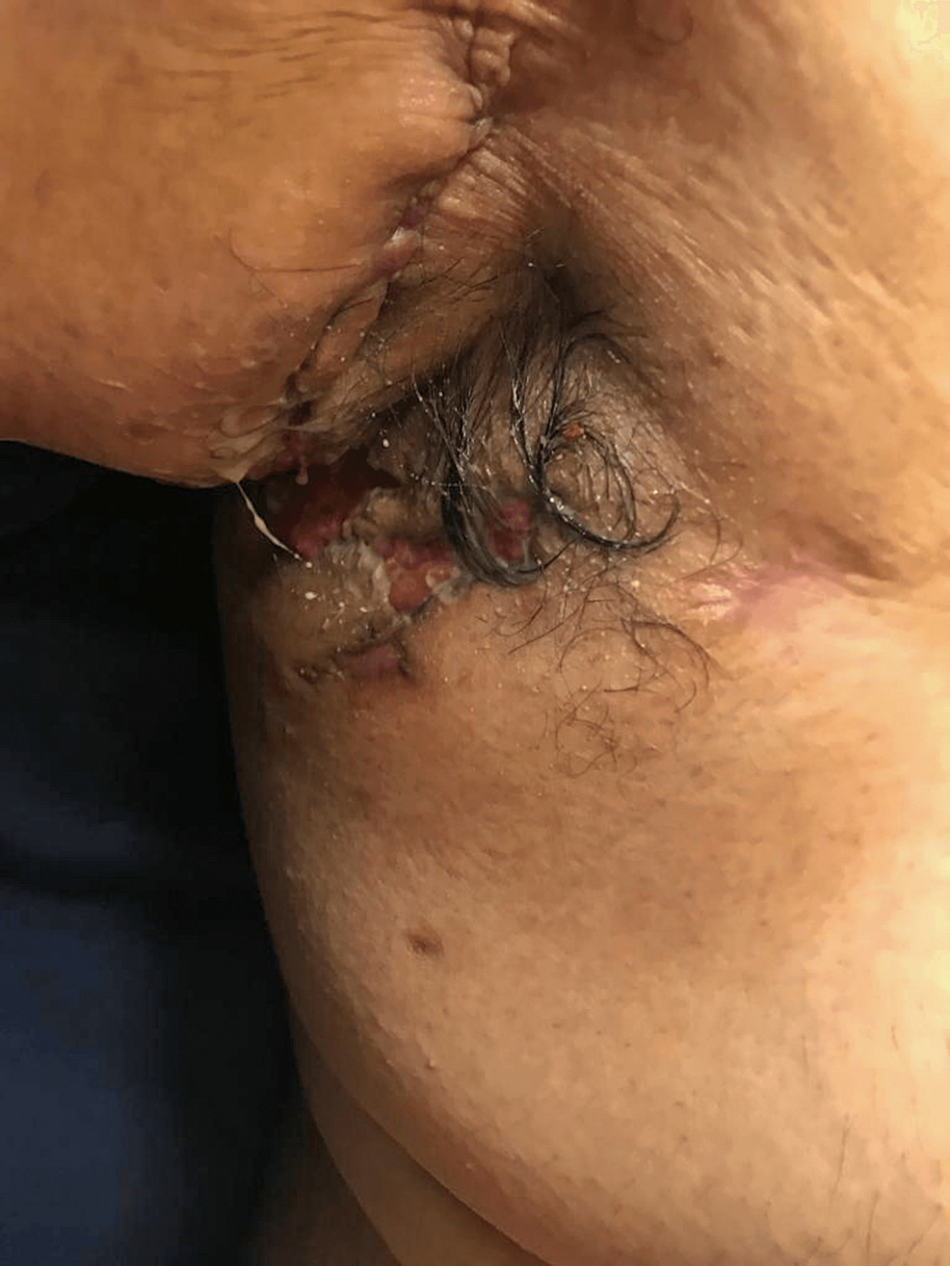
Hidradenitis suppurativa in the right axillary region

**Figure 3:**
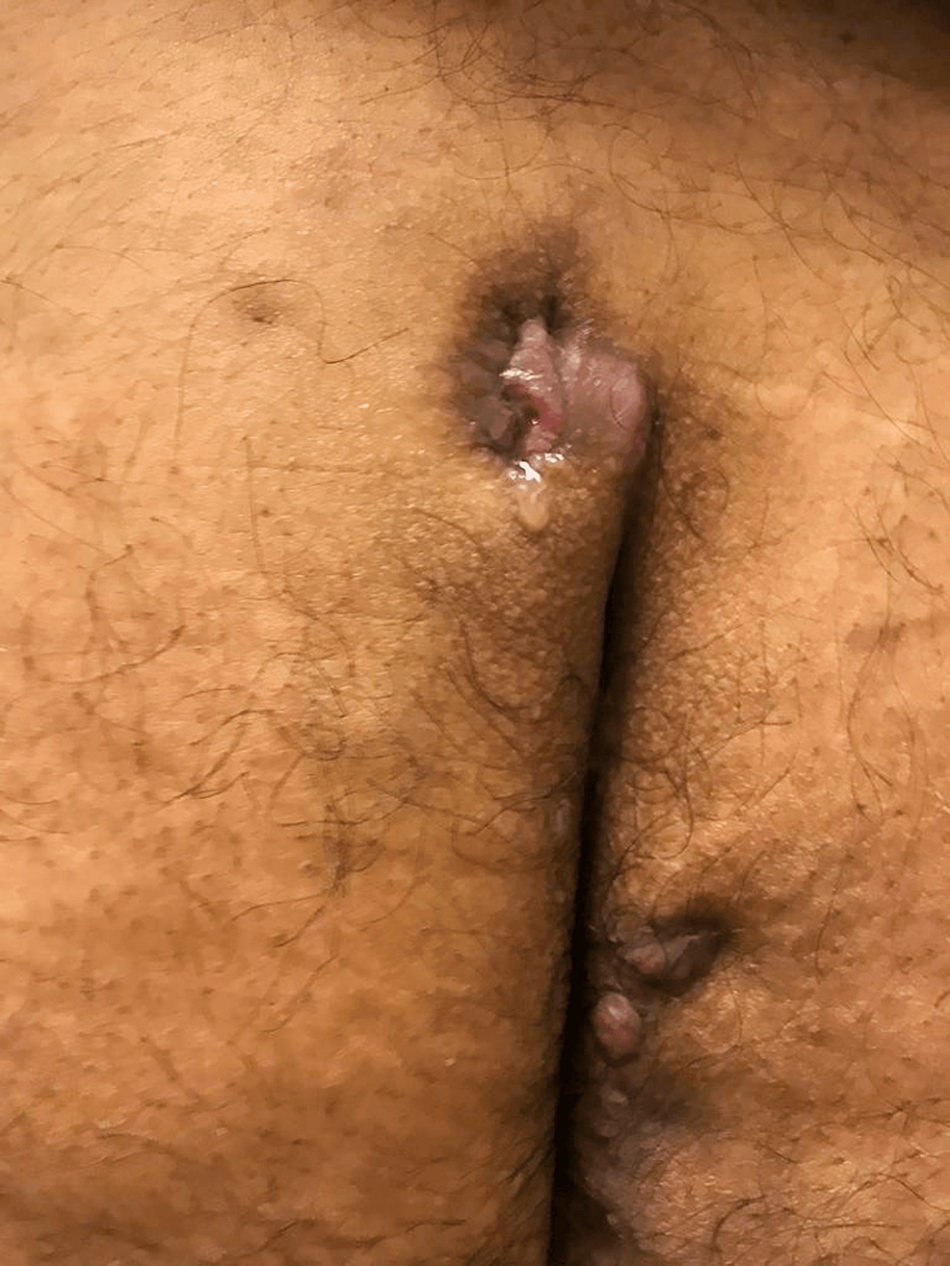
Pilonidal cysts - cysts in the intergluteal region

A 22-year-old obese Hispanic man was referred to the rheumatology outpatient clinic with worsening skin lesions in the face, scalp, axilla, lower abdomen, and sacral areas. Symptoms began at age 17. The patient initially developed severe acne of the face not responding adequately to medical treatment. The patient underwent an incision and drainage of the right cheek lesion, however, the acne recurred and was resistant to escalated medical treatment. A year later, he developed left axillary purulent skin inflammation (Figure 1), which, after two years, went on to affect the right axilla (Figure 2), gluteal and perineal area (Figure 3). Based on clinical findings, a diagnosis of FOT was made. 

The patient was evaluated by multiple specialists, including infectious disease, dermatology, plastic surgery, and endocrinology. Despite treatment with antibiotics and isotretinoin, the patient’s symptoms progressively worsened, and he was referred to the rheumatology service for possible biologic treatment.  

**Figure 4:**
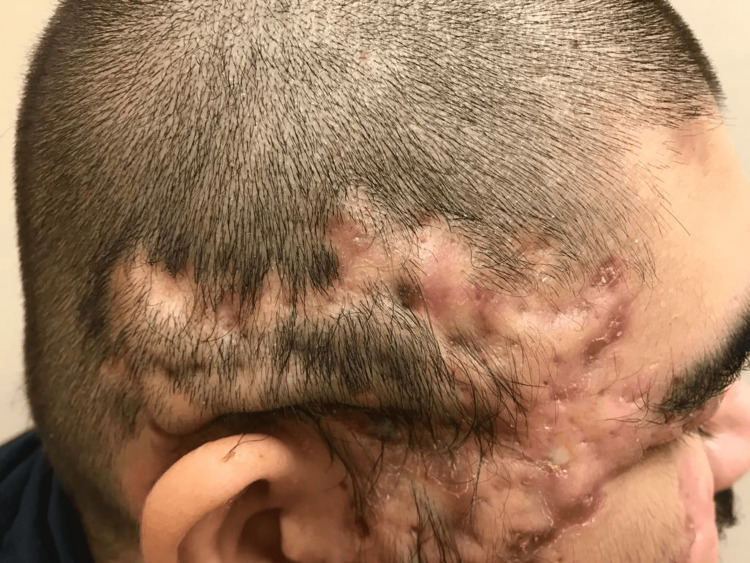
Dissecting cellulitis of the scalp - Scarring alopecia of the scalp

**Figure 5:**
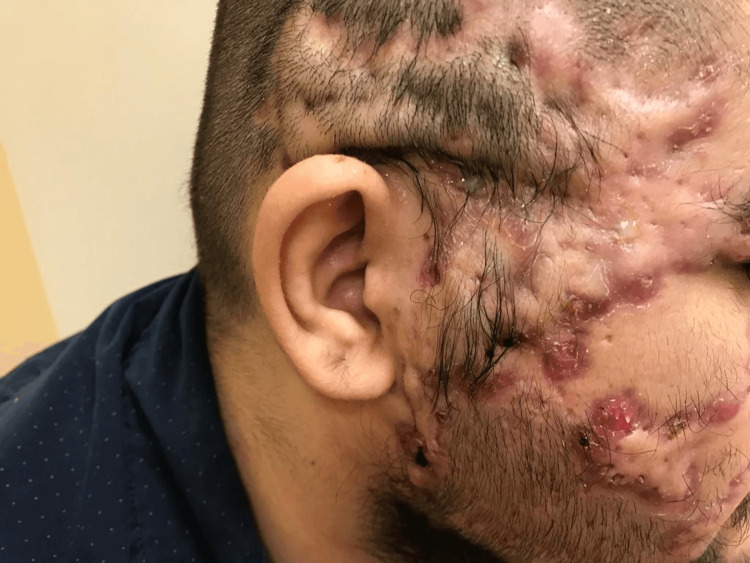
Dissecting cellulitis of the scalp - Alopecia, scarring of the scalp and face

The patient’s physical examination was remarkable for nodulocystic acne of the face with purulent drainage and burrowing abscesses consistent with AC. Scalp examination was remarkable for scarring alopecia (Figure 4) from previous lesions as well as interconnecting sinus tracts compatible with DCS. Some lesions of the face and scalp were tender to palpation and were oozing foul-smelling material (Figure 5). Examination of the axillae showed severe bilateral HP lesions with the right axilla affected area of 7x3.5 cm and with a 2x1.5 cm satellite lesion. The left axilla had a similar affected area of 7x3.5 cm with two smaller satellite lesions. The sacral region had a PC with active purulent discharge through an open sinus tract measuring 7 cm in depth. Lesions were painful, especially in the sitting position. Similarly infected and draining HS-like lesions were seen in the gluteal, abdominal pannus, perineum, and bilateral groin areas. There was no associated lymphadenopathy. The rest of the physical examination including cardiovascular, respiratory, neurologic, and musculoskeletal findings was unremarkable.

Laboratory studies revealed leukocytosis of 17,000/mm3 (reference values: 4800/mm3 - 10900/mm3); hemoglobin of 8.9 gm/dL (reference values: 11.6 gm/dL - 15.9 gm/dL), and hematocrit of 32.2% (reference values 34.1% - 47.5%), platelets of 532 k/mm3 (reference values: 146 k/mm3 - 388 k/mm3).  The sedimentation rate was elevated at 120 mm/h (reference values 2 mm/h - 30 mm/h) and C-reactive protein (CRP) was 11.1 mg/L (reference value <1.0 mg/dL). Liver function and renal function were within normal limits along with blood glucose levels.  

Multiple cultures were sent, including blood, and wounds from the gluteal, facial, and axillary lesions. Blood culture was negative, however, skin cultures of the gluteal lesions were positive for *Veillonella species* and *Anaerococcus prevotii.* Facial and axillary lesions were positive for *Proteus mirabilis*. During the disease course, he was managed with different regimens of antibiotics, including clindamycin, rifampin, trimethoprim-sulfamethoxazole, minocycline, and amoxicillin-clavulanate with incomplete response. Dapsone was tried with no improvement. Isotretinoin was associated with worsening symptoms. Surgery was recommended to be performed on multiple stages due to the lack of response to conservative medical treatment and secondary bacterial infections which precluded the use of tumor necrosis factor (TNF)-alpha blocker. 

The staged surgical procedures included excisional debridement of each axillary HP wounds complex, closure of bilateral axillary HP wounds, excisional debridement of bilateral temporal wounds, closure of right temporal wound with a combination of a large rotational scalp flap and placement of a full-thickness skin graft from the right supraclavicular area donor site, closure of left temporal wound with a combination of local scalp skin adjacent tissue transfer skin graft from the left supraclavicular area donor site, excisional debridement of glabellar hidradenitis, closure of the glabellar wound with a placement skin graft from the left supraclavicular donor site. Subsequently, he underwent excision of the lower abdomen, pubic, scrotum, bilateral groins HS, primary complex closure, and locoregional flaps. Surgical pathology was sent which reported granulation tissue and significant acute inflammation and negative for malignancy.

The patient is currently doing well; incisions in the face, abdomen, groin, and scalp have healed and closed. 

## Discussion

The coexistence of hidradenitis suppurativa, dissecting cellulitis of the scalp, acne conglobata, and pilonidal cyst constitute the Follicular Occlusion Tetrad (FOT). This tetrad was first coined in 1975, and, due to its rarity, no precise prevalence of the disease was reported in the literature. In a review article, 19 cases of follicular occlusion triad and four cases of follicular occlusion tetrad were found [2]. Of these, only two were complete cases of FOT and the other two were lacking DCS. The pathophysiology of FOT is not very well defined. However, it has been suggested that the four components share the same pathogenesis. 

The syndrome was first described as a follicular occlusion triad in 1956 by Pillsbury and Shelley [3]. Later on, pilonidal cyst was added [2], and follicular occlusion tetrad terminology was introduced. Very few cases were reported. In a literature review of syndromic HS, only four cases out of 82 had FOT. 

Our case report is intended to increase awareness in order to improve the ability to diagnose and appropriately manage this complicated medical entity.  In this review, we include the description of the four entities comprising FOT. 

Hidradenitis suppurativa (HS) 

HS is caused by poral occlusion involving predominantly the axillary, anogenital, postauricular area, and the gluteal and inframammary regions. It is hypothesized that the primary event in HS is follicular hyperkeratosis and follicular occlusion. This leads to keratinous plugging of the apocrine sweat duct, dilatation of the duct, and subsequent inflammation around the gland [4] with rupture of the follicle. While disease presentation varies, characteristic lesions are deep-seated nodules that expand to form abscesses and subsequently rupture and drain via sinus tracts and fistulae. The rupture of the follicule releases its contents, including keratin and bacteria, into the dermis. Ample research focuses on the role of inflammatory cytokines, like TNF alpha, interleukin (IL) 1 Beta, IL 2 as well as IL 12, IL 17, and IL 23 as key factors in developing HS along with genetic predisposition, environmental and hormonal factors, as well as pathogenic microorganisms. 

Many efforts have been made to create a useful and applicable tool to measure disease severity and treatment responsiveness. The first classification score was introduced by Hurley in 1989 [5]. It comprises three stages depending on the presence of sinus tract and scarring. The initial scoring system was not quantitative. Sartorius et al developed a more detailed scoring system that quantifies disease severity and grade of inflammation. However, it was more complex and time-consuming for physicians to use [6]. In 2018, the Severity Assessment of Hidradenitis Score (SAHS) [7] was introduced to be more easily applied and is designed mainly to differentiate between fistulas and other inflammatory or painful lesions other than fistulas. At presentation, our patient was more likely in stage 3, which is consistent with five or more regions involved, including four or more fistulas, ten or more non-fistula inflammatory lesions, and five or more flared boils. However, the pain severity was less than 7 on the numerical rating scale. 

Compared with patients without HS, patients with HS have a 65% higher risk of developing ankylosing spondylitis, a 44% higher risk of developing psoriatic arthritis and a 16% higher risk of developing rheumatoid arthritis** **(RA)** **[8]. 

The FDA-approved treatments for HS include the TNF-alpha blocker adalimumab. IL 23 inhibitor guselkumab has been used in the treatment of recalcitrant DCS, which was described in a patient with concomitant HS. Studies of HS have shown increased levels of IL 23 within macrophages in lesional dermis suggesting IL 23 inhibition as a potential therapeutic target. 

Acne conglobata (AC) 

Acne conglobata is a rare chronic inflammatory disorder of the skin with a very severe presentation resulting in scar formation and disfigurement. It can occur as a rapid deterioration of existing papular or pustular acne or a resurgence of dormant acne. The exact pathophysiology is still unclear; however, it is proposed to be an interplay of multiple components. All acne starts off as a micro comedo, transforms into comedones with follicular hyperkeratinization, and is hormonally influenced by increased sebum production. The gram-positive microaerophilic/anaerobic rod organism, *Cutibacterium acnes*, a skin commensal seen in sebaceous follicles, is said to set off an inflammatory trigger leading to rupture of the follicular wall, forming cystic acne. Furthermore, underneath the nodule, there is an intense inflammatory reaction with pus formation. Patients usually present with multiple comedones, pustular cysts, interconnected abscesses, and scars mainly distributed in the face, chest, bilateral shoulders, back, upper arm, buttocks, and thighs [9]. 

It is to be noted that while regular acne vulgaris can be treated with topical medications, acne conglobata can be extremely difficult to treat, especially when it is part of the follicular tetrad, and requires aggressive systemic treatment. Transdermal penetration of topicals and their systemic bioavailability is not clear, the reason why systemic medication is indicated in moderate to severe acne. The treatment recommendation for acne conglobata is a 20- to 28-week course of oral retinoids along with adjuvant topical retinoids. Oral retinoids like isotretinoin can sometimes lead to acne fulminans, an immunologically induced systemic response, resulting in severe disease with draining lesions, associated with systemic symptoms like fever, malaise, and weight loss. Initiation of adjuvant oral steroid therapy (oral prednisone 1 mg/kg/d) for the initial two to four weeks is recommended to prevent this response. In cases of acne fulminans, temporary discontinuation of isotretinoin, steroid initiation, and later reinitiating isotretinoin at a smaller dose to titrate up, followed by steroid tapering is recommended. Patients have to be on oral contraception while on retinoids. Tetracyclines, either doxycycline or minocycline 100 mg twice daily, are an alternative option; however, a combination with retinoids should be avoided as that can sometimes lead to pseudotumor cerebri. In purulent lesions, fluid aspirate can be sent for cultures; however, antibiotics, usually tetracyclines, are to be initiated while awaiting results. Reviewing literature showed that even though isotretinoin is superior to dapsone based on previous trials, dapsone can be used in refractory cases and in cases where isotretinoin is contraindicated, showing adequate results. Glucose-6-phosphate dehydrogenase (G6PD) level is to be obtained prior to dapsone administration, and routine laboratory monitoring while on treatment should be done to prevent its well-known side effects such as hemolytic anemia, methemoglobinemia, and agranulocytosis [10]. 

In a study done by Graham et al, it was found that live *Propionibacterium acnes* (now known as *Cutibacterium acnes*) stimulated keratinocytes to produce TNF-alpha and interleukin-1-alpha [11]. The pathophysiology behind using TNF-alpha inhibitors is that the inhibition of the cytokine cascade, of which TNF-alpha is a part, might benefit in reducing the inflammatory cascade. There have been at least a few case reports of acne conglobata reported in literature where adalimumab was used for refractory treatment and prompted resolution in 12 weeks. Adalimumab is FDA approved for the management of HS but not approved in AC or similar conditions that are part of the follicular tetrad. Infliximab is another TNF-alpha inhibitor that has been reported to be used in refractory disease, leading to a resolution of the lesions. Etanercept has also been reportedly used in cases of nodulocystic acne. All three have been reported to be used in cases of acne conglobata associated with synovitis, acne, pustulosis, hyperostosis, osteitis (SAPHO) syndrome. In presentations where they coexist with other conditions, forming the follicular tetrad, treatment can be even more difficult, and treatment with TNF-alpha inhibitors has been reported to have good outcomes. In conclusion, TNF-alpha inhibitors can be used in patients with recalcitrant acne [12,13]. External beam radiation and laser therapy have also been reportedly used in refractory cases. Surgical treatment options consist of surgical aspiration of fluctuant cystic nodules and sometimes excision of nodular lesions. Dermal fillers can be used later once inflammation resolves [14]. There have been a few case reports where laser treatment seems a promising therapeutic modality in the management of pilonidal sinus disease itself without performing any surgical interventions, especially in the initial stages of cyst formation where the treatment could be even more effective. 

Pilonidal cyst (PC) 

A pilonidal cyst or sinus has been reported as a common suppurative condition that affects young adults in the intergluteal region through the supposed embedding of hair tracts into a damaged epithelium, which secondarily has inflammation and infection [14]. Pilonidal derives its name from Latin *pilus* meaning “hair,” and *nidus* meaning “nest" [15]. Originally, an ectodermal remnant of the neural tube was thought to be the primary cause. However, it remains unclear whether the development of pilonidal disease is secondary to local pressure on the tissues, ruptured hair follicles, hypoxic tissue beds, or a congenital vulnerability of the natal skin [16]. Part of the etiology is explained due to natal cleft stretching while sitting or bending, leading to damage or breaking of the hair follicles and opening of a pore or “pit” [16]. Evaluation of pilonidal sinuses has shown that the cystic cavities within the sinus branch outward, and these are lined by chronic granulation tissue. The sinus lining is characterized by hemosiderin-laden macrophages and sometimes even foreign-body giant cells, reflecting the chronic inflammatory picture at play [17]. The spectrum of pilonidal disease presentation varies from a chronic cyst and/or sinus with persistent drainage and/or extensive subcutaneous tracts to the more acute presentation of an associated abscess. Clinically the lesion is asymptomatic until it becomes infected. The conservative approach is usually the standard of care, which includes reassurance and hygiene, as the disease may dissipate as the patient passes the fourth decade of life. However, when patients start complaining about acute pain, with swelling, erythema, and a tender lump in or near the natal cleft, the option for treatment includes needle aspiration, drainage without curettage of the cavity, or primary drainage and curettage of the cavity to remove any hair follicles or debris [16]. The choice of a particular surgical approach depends on the surgeon's familiarity with the procedure and perceived result in terms of low recurrence of sinus and quick healing of the resulting cavity or surgical wound [17].

The value of antibiotic therapy in an acute pilonidal abscess has not been clearly established in the absence of immunodeficiency or concurrent systemic illness. When there is a history of repeat abscesses, various surgical techniques can be considered to treat the chronic symptomatic pilonidal sinus, among them: radical excision, deroofing, debridement of the sinus tract followed by primary flap coverage, as well as endoscopic pilonidal sinus treatment [18]. The American Society of Colon and Rectal Surgeons recommends the elimination of hair from the gluteal cleft and surrounding skin by shaving or laser epilation for both acute and chronic pilonidal disease in the absence of abscess as a primary or adjunct treatment measure, with class 1C level of recommendation. Furthermore, surgical excision is the standard treatment for chronic pilonidal cyst and sinus disease and is generally divided into two categories: (1) excision of diseased tissue with primary closure (including various flap techniques) versus (2) excision with healing by secondary intention (including marsupialization) [16]. 

Dissecting cellulitis of the scalp (DCS) 

Dissecting cellulitis of the scalp, also known as perifolliculitis capitis abscendens et suffodiens or Hoffman disease, is a rare and severe chronic inflammatory disease of the pilosebaceous unit of the scalp. It manifests with perifollicular pustules, nodules, abscesses, and sinuses with multiple remission and relapses that evolve into scarring alopecia [18,19], it has a propensity for occurrence in African American men 20-40 years of age. Histologically, dissecting cellulitis resembles HS, with early lesions characterized by heavy infiltration of the follicular and perifollicular neutrophils, lymphocytes, histiocytes, and plasma cells, with granulomas, resulting in abscess formation in the dermis of the scalp and scarring and fibrosis see in later stages [20,21]. Differential diagnosis for DCS is broad, including tinea capitis, pseudopelade of Brocq, squamous cell carcinoma, metastatic Crohn’s disease, and erosive pustular dermatosis of the scalp. Dissecting cellulitis of the scalp, HS, and AC are differentiated mainly on their clinical presentation, with DCS affecting the scalp, HS affecting axillary and anogenital regions, and AC affecting the back, buttocks, and chest [19,21]. The diagnosis of DCS can usually be made clinically, however, culture should be performed to rule out secondary infection. In some cases, a biopsy may be necessary to confirm the diagnosis and rule out other forms of cicatricial alopecia if overlapping features are observed. 

Since data on treatment options for DCS are limited and there are no consensus guidelines on therapeutic approaches, treatment of DCS should focus on reducing inflammation and follicular occlusion and preventing secondary infection [22]. The systemic use of isotretinoin, dapsone, and TNF inhibitors (adalimumab and infliximab) has been described with varying degrees of efficacy. For those that are refractory to medical therapy, surgical excision of the scalp with split-thickness skin grafting has been effective [23]. Notwithstanding numerous treatment options for DCS, relapses are common and often result in significant psychological distress for patients. There is a report of an IL 23 inhibitor (guselkumab) used in the treatment of recalcitrant DCS, which was described in a patient with concomitant HS. Studies of HS have shown increased levels of IL 23 within macrophages in the lesional dermis, thus it was speculated that IL 23 may also be implicated in the pathogenesis of DCS [22]. 

## Conclusions

The clinical manifestations of follicular occlusion tetrad share a common pathogenesis involving multiple factors including inflammation, abnormal keratinization, and dysregulation of the immune system. Abnormal keratinization of hair follicles and subsequent obstruction combined with inflammation lead to neutrophilic abscesses and granulomas in the affected area. Immune dysregulation then causes increased production of pro-inflammatory cytokines, resulting in chronic inflammation and tissue damage. Treatment of the follicular occlusion tetrad is often challenging and requires long-term care from a multidisciplinary team. Stepwise therapy from topical or oral antibiotics to anti-inflammatory medications such as nonsteroidal anti-inflammatory drugs (NSAIDs), retinoids to immune modulators like TNF-alpha, IL 17, and IL 23 blockers, as well as surgical therapies, may be used. While retinoids may be effective in managing acne conglobata, their use in the management of other conditions of the tetrad, such as HS, is controversial. In addition to medical and surgical modalities, lifestyle modifications including weight loss, good skin hygiene, avoiding tight-fitting clothes, and avoiding shaving can play a role in management. In cases where medical and conservative approaches fail, surgical intervention may be necessary. 

In our patient, oral retinoids were ineffective and the lesions continued to progress and led to complicated secondary bacterial infections that grew different bacterial species. Due to the potential risk of sepsis with TNF-alpha blocker treatment, the patient instead underwent a series of surgeries that included radical excision of the lesions. Multiple excisional debridements and complex closures of the affected sites were performed by plastic surgery. In addition, smoking cessation and weight loss in our patient resulted in the resolution of their follicular occlusion tetrad and did not require additional medical treatment.
